# The effect of *Kappaphycus alvarezii* active fraction on oxidative stress and inflammation in streptozotocin and nicotinamide-induced diabetic rats

**DOI:** 10.1186/s12906-021-03496-8

**Published:** 2022-01-13

**Authors:** Evy Yulianti, Sunarti, Mae Sri Hartati Wahyuningsih

**Affiliations:** 1 Department of Biology Education, Faculty of Mathematics and Science, Universitas Negeri Yogyakarta, Yogyakarta, Indonesia; 2 Department of Biochemistry, Faculty of Medicine, Public Health and Nursing, Universitas Gadjah Mada, Yogyakarta, Indonesia; 3 Department of Pharmacology and Therapy, Faculty of Medicine, Public Health and Nursing, Universitas Gadjah Mada, Yogyakarta, Indonesia; 4 Herbal Medical Center, Faculty of Medicine, Public Health, and Nursing, Universitas Gadjah Mada, Yogyakarta, Indonesia

**Keywords:** Diabetes, H_2_O_2_, *K. alvarezii*, NFκB, NOX4, TNFα

## Abstract

**Background:**

High glucose concentration increases the glycation process which leads to oxidative stress and inflammation, that can cause complications in diabetes. Several medicinal plants have been used in the treatment of diabetes and its complications. One of them is *Kappaphycus alvarezii,* an algae that has known antidiabetic abilities. This study aimed to examine the effect of *K. alvarezii* active fraction on plasma hydrogen peroxide (H_2_O_2_) and *Tumor Necrosis Factor* α (TNFα) levels, renal NADPH oxidase 4 (NOX4) and *Nuclear Factor* κ *B* (NFκB) gene expressions.

**Methods:**

Active fraction was obtained from bioassay-guided fractionation with antiglycation ability. In vivo study was performed on twenty Wistar male rats. The level of H_2_O_2_ was measured using H_2_O_2_ Assay Kit, the Optical Density value measured using spectrophotometer at a wavelength of 405 nm. Plasma TNFα level was measured using ELISA. Renal NOX4 and NFκB gene expression was analyzed using qPCR.

**Results:**

Active fraction significantly reduced plasma H_2_O_2_ but not TNFα levels. Furthermore, renal NOX4 gene expression was lower in the diabetic rat group treated with active fraction compared to the untreated group but not NFκB gene expression.

**Conclusions:**

*K. alvarezii* active fraction has an activity to reduce plasma H_2_O_2_ as well as renal NOX4 gene expression. Therefore, this fraction could be developed as a potential candidate for diabetes treatment through oxidative stress mechanisms.

## Background

Diabetes is a disease characterized by high blood glucose levels or hyperglycemia, usually accompanied by disorders of carbohydrate, fat and protein metabolism [1]. The reduced absorption of glucose into muscles and adipose tissue causes chronic extracellular hyperglycemia, which results in tissue damage and complications [2]. The increase in blood glucose causes an imbalance in the production of free radicals and antioxidants. Oxidative stress and inflammatory conditions, especially those associated with obesity, will lead to insulin resistance, pancreatic beta cell dysfunction and vascular complications in type 2 diabetes [3].

Nephropathy, neuropathy, and retinopathy in patients with diabetes are the main microvascular complications induced by chronic hyperglycemia through several mechanisms, such as A*dvanced Glycation End Products (*AGEs) production, inflammation, and hyperglycemia-induced oxidative stress [4]. Very high levels of free radicals cause damage to vital cellular components such as proteins, membrane lipids, and nucleic acids, and ultimately lead to cell death [5].

Production of Reactive Oxygen Species (ROS) in the cytosol results from the activation of various enzymes, including NADPH oxidase (Nox), myeloperoxidase, xanthine oxidase, cytochrome p450 mono-oxygenase, cyclooxygenase and nitric oxide synthase (NOS). Nox is a source of cytosolic ROS in diabetes [6, 7]. The production of ROS is generated constitutively and mostly in the form of Hydrogen Peroxide (H_2_O_2_) [8]. The Nox family is a cellular enzymatic system that has the main function of producing ROS [6]. Nox4 is a NADPH-oxidase isoform that is expressed in the greatest amount in the kidneys [8, 9].

Nuclear factor kappa B (NFκB) is a cytoplasmic transcription factor of every cell and will move to the nucleus when activated [10]. NFκB plays an important role in the pathogenesis of diabetes-related vascular complications. Hyperglycemia activates NFκB which will trigger the expression of various adhesion molecules, chemokines and cytokines, including tumor necrosis factor-alpha (TNF-α), interleukin, transcription growth factor-beta (TGF-β), Bcl2 and other proinflammatory proteins [11].

Seaweed is one of the leading commodities in Indonesia. One of them is *K. alvarezii*. These algae contain an abundance of carbohydrates, proteins, alkaloids, glycosides, flavonoids, steroids, and phenolic compounds [12]. Petroleum ether and ethanol extracts showed the presence of reducing sugars, alkaloids, flavonoids and glycosides in *K. alvarezii*. This algae also contains terpenoids and steroids in small amounts. Several compounds have been identified from these algae, including chlorogenic acid, sinapic acid, hydroxylenzoic acid, gallic acid, phloroglucinol, vanillic acid, cinnamic acid, catechol and ferulic acid [13].

Previous study using diabetic rats has shown that the active fraction from this algae has antiglycation properties by reducing AGEs, namely glycated albumin (GA) and Nε- (carboxymethyl) lysine (CML) [14]. Phytochemicals inhibit the formation of AGEs through various pathways, including lowering glucose levels, inhibiting aldose reductase, metal chelation activity, hypolipidemic activity, free radicals scavenging, reducing AGEs cross-links, competing with reducing sugars to bind to proteins, and inhibiting TNF, which is involved in insulin resistance. The ability as antioxidants or chelating activities of phytochemicals cause inhibition of AGEs formation [15]. *K. alvarezii* is thought to be a rich source of antioxidants. This algae can also stimulate the wound healing process and act as an anti-inflammatory agent to prevent tissue damage because it has phenolic compounds and their derivatives [16, 17]. Accordingly, the aim of this study was to examine whether the active fraction from *K. alvarezii* can be developed as an alternative in diabetes treatment because of its antioxidant and anti-inflammatory properties.

In this study, rats were used as animal models because rat have a similar metabolism to humans. The selection of male rats was due to the fact that male rats were more easily induced to become diabetic using *streptozotocin* (STZ) and nicotinamide (NA). Most rodent studies examining effects of manipulations on β cell mass and function have traditionally focused only on male adults because they have the stronger diabetic phenotype compared to females.

## Methods

### Instruments and chemicals

This study used a centrifuge (Hitachi 18PR/5), High Resolution Mass *Spectrometry* (HRMS) using Thermo Scientific™ Dionex™ Ultimate 3000 RSLCnano UHPLC coupled with Thermo Scientific™ Q Exactive™ High Resolution Mass Spectrometer, and *CFX96* Touch Real-Time Polymerase chain reaction (PCR) (Bio-Rad). The chemicals used were *Streptozotocin* (STZ) (Nacalai Tesque, Inc.) and nicotinamide (NA), H_2_O_2_ Assay Kit (Elabscience), ELISA kit for TNFα (Bioassay Technology Laboratory), RNA later solution (Invitrogen), RNA Kit (Invitrogen), iScript™cDNA Synthesis Kit (Bio-Rad), Sso Fast™ EvaGreen® Supermix (Biorad), Eppendorf tube 1.5 mL, qPCR tube 200 μL (Bio-Rad), tip, NOX4 primer, NFκB primer, and β-actin primer.

### Plant and animals material

*K. alvarezii* samples were collected from Lombok Tengah, Nusa Tenggara Barat, Indonesia. Plant determination was carried out at the Basic Biology Laboratory, Faculty of Mathematics and Natural Sciences, Mataram University, Indonesia, with a Certificate of Identification no 03/UN18.7/LB/2019. Active fraction used in this study was obtained from previous study [14]. Wistar male rats (200 g, aged 8 weeks) were obtained from Bogor Life Science and Technology (BLST) Company, Bogor, Indonesia. The experiment procedures with animals were approved by Medical and Health Research Ethics Committee of the Faculty of Medicine, Public Health and Nursing, Universitas Gadjah Mada, Yogyakarta, Indonesia with Ref. No.: KE/FK/0564/EC/2019.

### Study design

The resource equation was used to calculate sample size. This study used four groups. Minimum sample was *n* = 10/*k* + 1 (10/4 + 1 = 3.5) and maximum sample is *n* = 20/*k* + 1 (20/4 + 1 = 6) with *k* = number of groups, and *n* = number of subjects per group [18]. This study used five Wistar male rats in each group. These rats were acclimatized for 1 week in the laboratory. Rats were placed in individual cages on the same shelf in the same room under the same conditions with temperature ranges from 25 to 28 °C with a 12/12-h lighting cycle. Feeding and drinking were done ad libitum. Feeding, cleaning of all cages, giving treatment and sampling were carried out together in close time for all rats*.*

There were some inclusion criteria for the rats used in this study, i.e. healthy rats, has a weight of about 200 g, 8 weeks old male rats, and the exclusion criteria were sick rats (dull appearance of hair, loss or baldness and lack of activity or inactivity, abnormal discharge of exudate from the eyes, mouth, anus or genitals), and there was a weight loss of more than 10% after a period of adaptation in the laboratory.

### Bioassay guided fractionation

The active fraction from *K. alvarezii* were obtained from a previous study [14] using bioassay guided fractionation. Previous studies have succeeded in separating four fractions through the bioassay guided fractionation method (fraction I-IV), which we then selected the most active fraction as the active fraction (fraction II). This active fraction was used for the in vivo testing. The active fraction was determined based on its antiglycation ability using BSA-Glucose methods. The active fraction with highest antiglycation ability was used for the in vivo study.

### Compound analysis with high resolution mass spectrometer (HRMS)

The compounds in the active fraction used in this study were analyzed using High Resolution Mass Spectrometer (HRMS) as described in previous study [14].

### In vivo test

Three diabetic rat groups were induced with *nicotinamide* (NA) as much as 230 mg/kg bw intraperitoneally followed by *streptozotocin* (STZ) (65 mg/kg) in citrate buffer (0.1 M, pH 4.5) after fasting overnight. Nondiabetic control rats group was injected with citrate buffer (pH 4.5). Diabetic rats were divided into three groups: diabetic rats group, diabetic rats with 11 mg/kg BW *K. alvarezii* active fraction administration, and diabetic rats with 16.5 mg/kg BW *K. alvarezii* active fraction that was administered every day for 4 weeks. Samples of blood were collected from the retro orbital plexus after fasting overnight. Rats were fasted overnight before the sacrifice procedure and anesthetized using ketamine at a dose of 100 mg/kg BW IP. Rats were euthanized using cervical dislocation method. Kidneys of rats were collected in a tube containing RNA later solution and stored at − 80 °C.

### Plasma H_2_O_2_ and TNFα level

The level of plasma H_2_O_2_ was measured using H_2_O_2_ Assay Kit and the Optical Density value measured using spectrophotometer at a wavelength of 405 nm. Plasma TNFα levels were measured using ELISA method. Optical density (OD) values ​​at 450 nm wavelength were converted to TNFα levels using CurveExpert software.

### Renal NOX4 and NFκB gene expression

Rat kidneys (30 mg) were used to obtain RNA. The thermal cycling conditions for cDNA synthesis were incubation of 5 min at 25 °C, 30 min at 42 °C, 5 min at 85 °C, and finally at 4 °C, with the number of cycle was 1 cycle. Renal NOX4 and NFκB gene expression were analyzed with PCR conditions of 30 s at 95 °C, 5 s at 95 °C, 5 s at 55 °C, and finally 2–5 s/step at 65–95 °C for melting curve. The number of cycles was 40 cycles. The primer sequences for NOX4 gene were F 5′ GCTTGTTGAAGTATCAAACCAAT 3′ and R 5′ TCCAGAAATCCAAATCCAGGT 3′, NFκB F 5’AGGACCAGGAACAGTTCGAA3’ and R 5’CAGGTTCTGGAAGCTATGGAT3’ and the beta actin F 5′ TGTCACCAACTGGGACGATA 3′ and R 5′ ACCCTCATAGATGGGCACAG 3′ for reference. The level of gene expression was measured using relative quantification, with the 2^-∆∆CT^ arithmetic formula [19].

### Statistical analysis

Shapiro-Wilk test was used to analyze the normality of the data. Data with normal distribution were tested using paired T test and one-way ANOVA, followed with Duncan post hoc analysis, and the data with non-normally distribution were tested using Wilcoxon and Kruskal Wallis test. A *p* < 0.05 was considered statistically significant using SPPS 22 (IBM Corporation, Armonk, NY, USA).

## Results

1. **Plasma H**_**2**_ **O**_**2**_ **Level**

The H_2_O_2_ levels in the group of diabetic rats with *K. alvarezii* active fraction administration decreased 65.33 and 66.71% at a concentration of 11 and 16.5 mg/kg BW, respectively (*p* < 0.05). The H_2_O_2_ levels in the group of diabetic rats without the fraction also decreased by 9.82%, but this decrease was not statistically significant (*p* > 0.05).

**Fig. 1:**
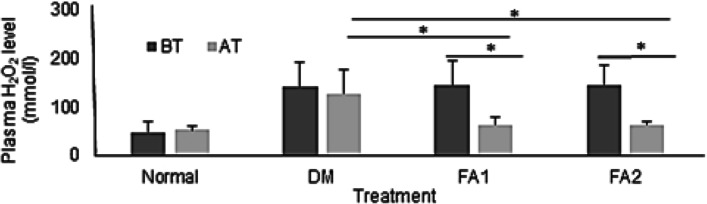
Plasma H_2_O_2_ levels. BT = before treatment. AT = after treatment. Normal = group of non-diabetic rats. DM = group of diabetic rats without the *K. alvarezii* fraction. FA1 = group of diabetic rats with the *K. alvarezii* fraction 11 mg/kg BW. FA2 = group of diabetic rats with the *K. alvarezii* fraction of 16.5 mg/kg BW. * = *p* < 0.05

Based on paired T Tests, administration of the *K. alvarezii* fraction was able to significantly reduce H_2_O_2_ levels (*p* < 0.05), to reach the same level as the non-diabetic group of rats (Fig. 1).

2. **Plasma TNFα Level**

**Fig. 2:**
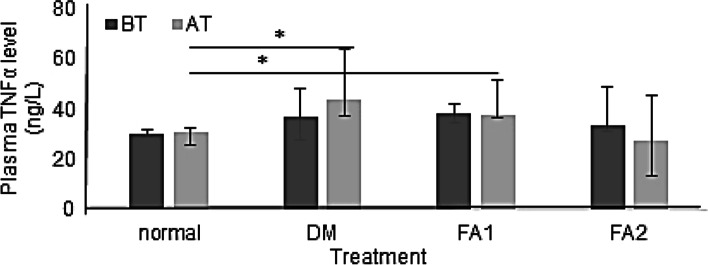
Plasma TNFα levels. BT = before treatment. AT = after treatment. Normal = group of non-diabetic rats. DM = group of diabetic rats without the *K. alvarezii* fraction. FA1 = group of diabetic rats with the *K. alvarezii* fraction 11 mg/kg BW. FA2 = group of diabetic rats with the *K. alvarezii* fraction of 16.5 mg/kg BW. * = *p* < 0.05

Based on Wilcoxon tests, TNFα levels before and after treatment did not show significant differences in all groups of rat (*p* > 0.05). TNFα levels in the group of diabetic rats with the *K. alvarezii* fraction of 16.5 mg/kg BW decreased by 17.14%, while the group of diabetic rats with the *K. alvarezii* fraction of 11 mg/kg BW decreased by 1.26%. Figure 2 shows an increase in TNFα levels due to hyperglycemia conditions in all groups of diabetic rats before administration of the *K. alvarezii* fraction. TNFα levels in the group of diabetic rats untreated with the *K. alvarezii* fraction were the highest among the other groups of rats.

3. **Renal NOX4 Gene Expression Analysis**

**Fig. 3:**
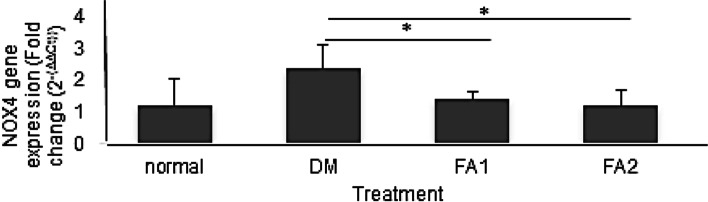
Renal NOX4 gene expression. Normal = group of non-diabetic rat. DM = group of diabetic rats without the *K. alvarezii* fraction. FA1 = group of diabetic rats with the *K. alvarezii* fraction 11 mg/kg BW. FA2 = group of diabetic rats with the *K. alvarezii* fraction of 16.5 mg/kg BW. * = *p* < 0.05

Renal NOX4 gene expression showed an increase in group of diabetic rat (Fig. 3). ANOVA test results showed a significant difference in the four groups of rats. Duncan’s post hoc test showed that the diabetic rat group differed significantly from the other three groups of rats. The group of diabetic rats without the administration of *K. alvarezii* fraction was significantly increased (*p* < 0.05) by 2.38 ± 0.72 times. The NOX4 gene expressions in groups treated with the *K. alvarezii* fraction were lower at concentration of 11 mg/kg BW (1.41 ± 0.21 times) and 16.5 mg/kg BW (1.20 ± 0.48 times) compared to the group of diabetic rats without treatment.

The NOX4 gene expression showed the highest results in the group of diabetic rats without *K. alvarezii* fraction compared to non-diabetic rats (Fig. 3). Giving *K. alvarezii* fraction can make NOX4 gene expression lower in both concentrations of 11 mg/kg BW and 16.5 mg/kg BW.

4. **Renal NFκB Gene Expression Analysis**

Duncan’s post hoc test showed a significant difference between the nondiabetic rats group and the diabetic rats group. The expression of NFκB gene showed an increase, in the group of diabetic rats without the *K. alvarezii* fraction administration (2.85 ± 0.83 times). Expressions of NFκB gene in the group of diabetic rats with *K. alvarezii* fraction of 11 mg/kg BW and 16.5 mg/kg BW administration were still higher than the group of nondiabetic rats (2.65 ± 1.87 times and 2.65 ± 0.94 times, respectively).

**Fig. 4:**
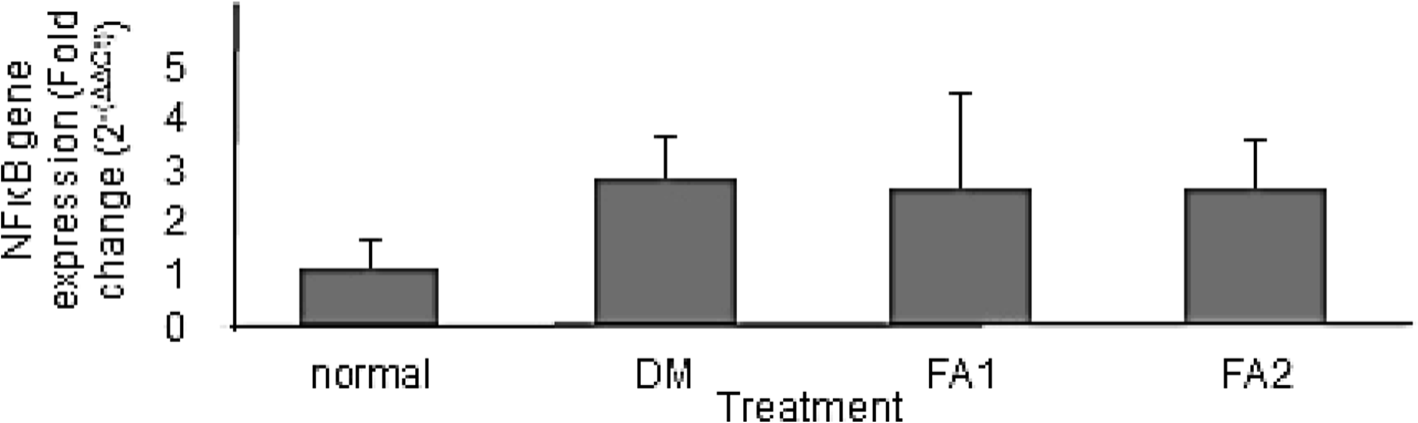
Renal NFκB gene expression. Normal = group of non-diabetic rat. DM = group of diabetic rats without the *K. alvarezii* fraction. FA1 = group of diabetic rats with the *K. alvarezii* fraction 11 mg/kg BW. FA2 = group of diabetic rats with the *K. alvarezii* fraction of 16.5 mg/kg BW

The four groups did not show a significant difference (*p* > 0.05) as shown in Fig. 4, however, there was a decrease in the expression of NFκB gene with the administration of *K. alvarezii* algae fraction. The highest expression of NFκB gene was in the group of diabetic rats without administration of *K. alvarezii* fraction.

5. **High Resolution Mass Spectrometry (HRMS) Analysis**

HRMS was used to identify some compounds in the active fraction of *K. alvarezii*, used in this study, some of which have been known to have antioxidant and anti-inflammation activity (Table 1).

**Table 1:** Results of analysis of active fraction content from *K. alvarezii* with antioxidant and anti-inflammation ability using HRMS

No	Name	Formula	Molecular Weight	RT [min]	Area (Max.)
1	Eicosapentaenoic acid	C_20_H_30_O_2_	302.224	25.373	32,214,931.15
2	Cinnamic acid	C_9_H_8_O_2_	148.052	3.402	4,936,572.72
3	Thymol	C_10_H_14_O	150.104	3.375	4,872,277.27
4	Docosahexaenoic acid ethyl ester	C_24_H_36_O_2_	373.298	27.731	547,049.777
5	Tyramine	C_8_H_11_NO	137.084	3.835	520,306.362
6	(E)-Ferulic acid	C_10_H_10_O_4_	194.058	17.175	362,404.05
7	Vanillic acid	C_8_H_8_O_4_	168.0421	12.886	304,421.64

Some of the data have been published [14]

## Discussion

Hyperglycemic conditions cause an increase in AGEs. Large amounts of ROS are generated during the formation of AGEs, and oxidized AGEs activate the RAGE receptors to stimulate Nox, which contributes to ROS production in diabetes [20, 21]. This condition improves oxidative stress [22] through the production of high ROS which can interfere with mitochondrial function and increase the activity of the Nox enzyme system. Nox produces intracellular ROS by transferring electrons from NADPH across the cell membrane and combining them to molecular oxygen to produce O_2_^• -^ [23]. In diabetes mellitus, the main source of oxidative stress is mitochondria. During oxidative metabolism in mitochondria, the used oxygen components are converted into water, and the remaining oxygen is converted into oxygen free radicals (O^•^) which are important ROS that will be converted to ONOO^−^, OH and H_2_O_2_ [24].

This process description is in accordance with the results of this study, in which H_2_O_2_ levels increased almost three times in the diabetic rats group compared to the non-diabetic group. Renal NOX4 gene expression increased in the group of diabetic rats without the active fraction in this study. Nox is the main source of ROS in all parts of the kidney [11]. Nox4 was first identified as NADPH oxidase which is highly expressed in the kidney with the main product being H_2_O_2_ [12].

Previous study has shown that active fraction of *K. alvarezii* with antiglycation activity could reduce plasma glycated albumin and Nε- (carboxymethyl) lysine (CML) levels as well as renal RAGE gene expression [14]. This causes the interaction between AGEs and RAGE receptors to decrease, so that the production of ROS also decreases. Giving active fraction of *K. alvarezii* could reduce H_2_O_2_ levels and NOX4 gene expression in the group of diabetic rats. The decrease in NOX4 activity was accompanied by a decrease of H_2_O_2_ levels. In addition, the active fraction of *K. alvarezii* contains antioxidant compounds (Table 1).

The TNFα levels after 4 weeks of treatment differed significantly between the groups of non-diabetic and diabetic rats as well as diabetic rats with an active fraction of 11 mg/kg BW. Meanwhile, TNFα levels in the group of diabetic rats with an active fraction of 16.5 mg/kg BW were not significantly different from the group of untreated diabetic rats. The TNFα levels in the group of diabetic rats without the active fraction were highest than the other groups.

The expression of the NFκB gene in the group of diabetic rats without the active fraction was significantly different compared to the group of non-diabetic rats after 4 weeks of treatment. The expressions of the NFκB gene were still high both in the diabetic rat groups by administering the active fraction. Hyperglycemia increases the expression of the NFκB gene and this pro-inflammatory agent can lead to insulin resistance in adipose tissue [14, 25]. Higher levels of NFκB have been reported in diabetic rat target organs such as the retina, heart, and kidneys [26]. Inflammation can be triggered by increasing ROS in diabetes mellitus.

ROS activates NFκB, which is a transcription factor that regulates the expression of proinflammatory genes or cytokines such as TNFα, IL-6 and C-Reactive Protein [27]. The H_2_O_2_ levels in the group of diabetic rats in this study increased, as did TNFα levels. H_2_O_2_ is able to induce kinases, such as Ras, mitogen-activated protein kinase (MAPK), and NF-kB-inducing kinase (NIK), regulating the activity of the IKK complex. The increase in IKK activity was associated with the phosphorylation of Ser180 and Ser181 on the activation of the IKKα and IKKβ loops and with the dimerization of IKKγ proteins through the formation of disulfide bonds. Activation of IKK results in translocation of the NFκB dimer to the nucleus [28]. NOX4-mediated oxidative stress can increase the dissociation of the NF-κB/IκB complex, so that NF-κB can move into the nucleus, further activating the transcription factor NFκB [29, 30]. NFκB then increases the transcription of various genes encoding inflammatory mediators, such as TNFα [31].

In this study, although the levels of H_2_O_2_ in the group of diabetic rats decreased by giving the active fraction, the levels of TNFα and expression of the NFκB gene remained high. This can be due to the activity of NFκB was not only affected by H_2_O_2_, but also from other pathways. Currently, there are three pathways that can be recognized to activate NF-κB: canonical, non-canonical, and atypical IκK independent pathways. NF-κB is naturally inhibited by IκB. The activation signal (binding of TNFα, IL-1α, LPS, CD40, Lymphotoxin, UV, HER2 / Neu, H_2_0_2_, or other ligands to their receptors) causes phosphorylation of IκB by IκB kinase (IκK). This triggers the degradation of IκB through the ubiquitin (Ub) system where the target molecule is covered by a chain of ubiquitins to be degraded by the 26S proteasome. Unbound free NF-κB can then be translocated to the nucleus and activate transcription [32].

High plasma glucose levels in the short term, alone or in combination with other risk factors, lead to the accumulation of NFκB-mediated transcription activation [33]. In this study, the high TNFα level itself causes the activation of NFκB. Table 1 shows the results of analysis of active fraction content from *K. alvarezii* with antioxidant and anti-inflammation ability using HRMS. Some of the data have been previously published [14].

Ferulic acid and vanillic acid are phenolic compounds present in the active fraction of red algae. These compounds have anti-inflammatory, antioxidant and antidiabetic properties. Ferulic acid has an electron donor group on the benzene ring (3-methoxy and 4-hydroxyl), so it acts as an antioxidant [34]. Cinnamic acid and its derivatives are the main group of phenolic acids which have antioxidant, anti-inflammatory, and anticancer activity [35]. Another compound in the active fraction that acts as an antioxidant is tyramine (4-hydroxyphenethylamine), a monoamine formed from the amino acid tyrosine. This compound has various biological properties, such as antioxidant, α1-adrenergic agonist and monoamine oxidase [36].

Tomobe et al. [37] showed that consumption of DHA ethyl ester (DHAEt) can provide anti-inflammatory effects. DHA decreases the expression of genes involved in the NFκB pathways (MAPK, AKT1, and NFκB), in addition, this compound can also decrease TNFα expression [38]. Another compound with anti-inflammatory activity is thymol. Thymol inhibits the phosphorylation of IκBα, NF-κB p65, ERK, JNK, and p38 MAPKs [39].

Additionally, eicosapentaenoic acid (EPA) reduces the incidence of increased palmitate-induced inflammatory gene expression and NFκB phosphorylation in 3 T3-L1 adipocytes. Diets containing EPA can reduce the increase of p-JNK level and phospho-p65 NFκB in adipose tissue [40]. Although the active fraction of red algae contains anti-inflammatory compounds, it has not been able to reduce inflammation that occurs in the group of diabetic rats treated with this active fraction. Active fraction has the ability to inhibit the glycation process and reduce oxidative stress but has no anti-inflammatory properties [41].

## Conclusions

In conclusion, *K. alvarezii* active fractions could significantly reduce plasma H_2_O_2_ level by 65.33 and 66.71% at concentrations of 11 and 16.5 mg/kg BW, respectively, but not plasma TNFα level. In addition, NOX4 gene expressions in the diabetic group of rats treated with these fractions were also lower than in the diabetic rats group without treatment but not NFκB gene expressions.

## Data Availability

All data generated or analyzed during this study are included in this published article. Raw data of this study can be asked to the corresponding author if needed.

## Abbreviations

AGE: *Advanced Glycation End Products*
CML: *Nε-(carboxymethyl) lysine*
H_2_O_2_: Hydrogen peroxide
IKK: *IkappaB* kinase
IL: Interleukin
JNK: *c-Jun N-terminal kinase*
MAPK: *Mitogen Activated Protein Kinase*
NA: *Nicotinamide*
NADPH oxidase: *Nicotinamide Adenine Dinucleotide Phosphate-Oxidase*
NFκB: *Nuclear factor kappa* B
Nox: NADPH oxidase
PKC: Protein Kinase C
RAGE: Receptor of *Advanced Glycation End Products*
ROS: *Reactive Oxygen Species*
STZ: *Streptozotocin*
TNF α: *Tumor necrosis factor-α*
